# lncRNA pair as candidate diagnostic signature for colorectal cancer based on the within-sample relative expression levels

**DOI:** 10.3389/fonc.2022.912882

**Published:** 2022-08-17

**Authors:** Ouxi Wang, Di Shi, Yaqi Li, Xiaoyan Zhou, Haidan Yan, Qianlan Yao

**Affiliations:** ^1^ Department of Pathology, Fudan University Shanghai Cancer Center, Shanghai, China; ^2^ Department of Oncology, Shanghai Medical College, Fudan University, Shanghai, China; ^3^ Institute of Pathology, Fudan University, Shanghai, China; ^4^ Department of Colorectal Surgery, Fudan University Shanghai Cancer Center, Shanghai, China; ^5^ Department of Bioinformatics, Fujian Key Laboratory of Medical Bioinformatics, School of Medical Technology and Engineering, Fujian Medical University, Fuzhou, China

**Keywords:** long non-coding RNA, colorectal cancer, biomarker, relative expression analysis, diagnostic

## Abstract

**Background:**

Early diagnosis of colorectal cancer could significantly improve the prognosis and reduce mortality. However, indeterminate diagnosis is often met in pathology diagnosis in biopsy samples. Abnormal expression of long non-coding RNA (lncRNA) is associated with the initiation and progression of colorectal cancer. It is of great value and clinical significance to explore lncRNAs as candidate diagnostic biomarkers in colorectal cancer.

**Methods:**

Based on the within-sample relative expression levels of lncRNA pairs, we identified a group of candidate diagnostic biomarkers for colorectal cancer. In addition, we validated it in independent datasets produced by different laboratories and different platforms. We also tested it in colorectal cancer tissue samples using quantitative real-time polymerase chain reaction (RT-qPCR).

**Results:**

A biomarker consisting of six lncRNA pairs including nine lncRNAs was identified for the diagnosis of colorectal cancer. For a total of 950 cancer samples and 247 non-cancer samples, both of the sensitivity and specificity could achieve approximately 90%. For adenoma samples, the accuracy could achieve 73%. For normal tissues from inflammatory bowel disease patients, 93% (14/15) were correctly classified as non-cancer. Furthermore, the lncRNA pair biomarker showed excellent performance in all clinical stages; even in the early stage, the accuracy could achieve 87% and 82% in stage I and II. Meanwhile, the biomarker was also robust to the microsatellite instability status. More importantly, we measured the biomarker in 35 colorectal cancer and 30 cancer-adjacent tissue samples using quantitative real-time polymerase chain reaction (RT-qPCR). The accuracy could achieve 93.3% (70/75). Specially, even in early-stage tumors (I and II), the accuracy could also achieve 90.9% (30/33). The enrichment analysis revealed that these lncRNAs were involved in highly associated cancer pathways and immune-related pathways. Immune analysis showed that these marker lncRNAs were associated with multiple immune cells, implying that they might be involved in the regulation of immune cell functions in colorectal cancer. Most of the biomarker lncRNAs were also differentially expressed between the mutant group and wild-type group of colorectal cancer driver genes.

**Conclusion:**

We identified and validated six lncRNA pairs including nine lncRNAs as a biomarker for assisting in the diagnosis of colorectal cancer.

## Introduction

Colorectal cancer (CRC) is the third most commonly diagnosed and the third most leading cause of mortality cancer worldwide (1). Early detection of CRC can significantly improve the prognosis and reduce the mortality of patients. However, at the early stage, only approximately 4/10 of patients with CRC could be diagnosed (2).

Currently, the established non-invasive tests usually have low sensitivity and a positive predictive value. The sensitivity of the fecal immunochemical test could only reach 79%. Meanwhile, the sensitivity of another methylation-based molecular marker Septin9 is only 48% (3). The current tumor serum protein biomarkers, such as CEA, CA19-9, and CA12-5, are not recommended for early diagnosis of CRC because of their low sensitivity (4). Biopsy sampling with less invasive techniques, colonoscopy, is the gold standard method for CRC screening. However, previous studies indicate that up to 8% of all CRCs are diagnosed after a colonoscopy that found no cancer (5). In addition, indeterminate diagnosis is often met in pathology diagnosis in biopsy samples (6, 7). Moreover, the biopsy location might be inaccurate, which might lead to inaccurate sampling of adjacent non-tumor tissues and reduce the diagnosis performance (8). However, previously reported diagnostic signatures are usually obtained by taking tumor-adjacent normal tissues as the normal samples. Therefore, these signatures cannot classify CRC-adjacent normal tissues that are not accurately sampled as CRC (9). Considering that the adjacent non-tumor colorectal tissues of CRC patients might have some molecular characteristics of CRC, it is possible to develop a signature to discriminate CRC (including CRC-adjacent tissues) from the tissues of non-tumor individuals.

Long non-coding RNA (lncRNA), a kind of RNA with length greater than 200 nt and the lack of protein-coding capacity, is involved in crucial regulatory processes such as apoptosis, cell proliferation, and immune regulation (10, 11). Clear accumulating evidence has shown that abnormal expression of lncRNA is associated with the occurrence and development of a variety of cancers, including CRC, and has a great clinical application value (11, 12). Moreover, lncRNA expression is tissue-specific. Therefore, it is of great value and clinical significance to explore lncRNA as early diagnostic biomarkers in CRC.

Previous marker screening methods are usually based on the absolute expression value of genes, which is sensitive to batch effects and hardly applicable to an individualized diagnosis (13). It has been reported that within-sample relative expressions of genes are robust to systematic biases and interindividual biological variations and tends to be highly stable in specific normal human tissues but widely disturbed in the corresponding cancer tissues (14). Therefore, it is reasonable and worthwhile to identify the candidate lncRNA diagnostic biomarkers of CRC based on the within-sample relative expression levels of lncRNAs.

In this study, six pairs of lncRNA were identified as candidate early diagnostic biomarkers for CRC based on their within-sample relative expressions. The performance was then validated in additional validation datasets. The lncRNA pair biomarker showed excellent performance in all clinical stages, even in the early stage, and robust to the microsatellite instability (MSI) status. This biomarker would be an effective candidate diagnostic biomarker of CRC.

## Materials and methods

### Data collection and preprocessing

The lncRNA expression profiles of colorectal tissues were collected and downloaded from the Gene Expression Omnibus repository (GEO) database (https://www.ncbi.nlm.nih.gov/geo/), Sequence Read Archive (SRA) database (https://www.ncbi.nlm.nih.gov/sra/), The Atlas of Noncoding RNAs in Cancer (TANRIC) database (https://www.tanric.org), The Cancer Genome Atlas (TCGA) database (https://portal.gdc.cancer.gov/), and UCSC Xena (http://xena.ucsc.edu/). It is worth mentioning that the normal tissues used in this study were from GTEx (https://www.gtexportal.org/), all from the autopsy samples of healthy human donors. The detailed information is listed in **Table 1**, **Table 3**, and **Table 4**. Processed matrix data are directly used for the data from the microarray platform in the GEO database. Raw counts were downloaded from UCSC Xena and converted to the fragments per kilobase of transcript per million mapped reads (FPKM) value. The FPKM of the lncRNA profile of the TCGA colorectal dataset was obtained from the TANRIC database. For RNA-seq data from the SRA database, “.sra” data were firstly converted to fastq using the fastq-dump tool and preprocessed using Trimmomatic software (15) and were then aligned to reference human genome GRCh39 by hisat2 (16). Read counts for each transcript were calculated by featureCounts (17) and were then converted to the FPKM value. For data from TANRIC, SRA, and UCSC Xena, the Ensemble gene IDs were converted to Entrenz ID. For data from the microarray platform in GEO, each probe was converted to an ID according to the corresponding platform file. The information for each lncRNA, such as Entrez ID and official gene symbol, was downloaded from the LNCipedia (18). Based on the collected information, we got the Entrez ID and RNA types for each probe. Those probes mapped to multiple genes were discarded. If multiple probes were mapped to the same lncRNA, the expression value of the lncRNA was defined as the arithmetic mean of the values of the mapped probes. The clinical information of CRC from TCGA was also downloaded.

**Table 1:** The training datasets used in this study.

Dataset	Platform	Normal	Adenoma	Cancer	Cancer adjacent

**GSE76987**	RNA-seq GPL11154	–	20	–	–
**GSE95132**	RNA-seq GPL16791	–	–	10	10
**GSE87096**	RNA-seq GPL11154	–	–	6	6
**GSE101588**	RNA-seq GPL11154	–	–	17	–
**GSE121842**	RNA-seq GPL17303	–	–	3	3
**GSE92914**	RNA-seq GPL18573	–	–	6	3
**GSE50760**	RNA-seq GPL11154	–	–	18	18
**GTEX**	RNA-seq	155	–	–	–
**GSE8671**	MicroArray GPL570	–	32	–	
**GSE117606**	MicroArray GPL25373	–	–	40	–
**Total**		155	52	100	40

### Identify candidate long non-coding RNA pairs for diagnosis of colorectal cancer

For each sample, the expression levels of lncRNAs were firstly transformed to the relative expression orderings (REOs) of lncRNAs. For each lncRNA pair, *L_i_* and *L_j_* (*i* = 1…*n*, *j* = 1…*n*, and *i* ≠ *j*), and *e_i_* and *e_j_* represented the expression level of *L_i_* and *L_j_*, respectively; *n* represented the total number of lncRNAs. Then, the REO of *L_i_* and *L_j_* in a sample was *e_i_* > *e_j_* or *e_i_* < *e_j_*. If the within-sample REO is kept in more than 70% of cancer (non-cancer) tissue samples, this lncRNA pair is defined as consistent lncRNA pairs in CRC (non-cancer) samples. If the expression pattern of a consistent lncRNA pair is *e_i_* >*e_j_* (or *e_i_* <*e_j_*) in CRC samples and is *e_i_* <*e_j_* (or *e_i_* >*e_j_*) in non-cancer samples, this lncRNA pair is defined as the reversal lncRNA pair between cancer and non-cancer samples. Furthermore, geometric mean (avgR_ij_) is calculated to evaluate the reversal degree for each reversal lncRNA pair as follows, avgR_ij_= $\sqrt{\text{mean}\left[\text{Rij}\left(\text{cancer}\right)\right]\times \text{mean}\left[\text{Rij}\left(\text{non}-\text{cancer}\right)\right]}$ ; R_ij_=|R_i_-R_j_|, in which R_i_ and R_j_ represent the ranks of lncRNA *L_i_* and *L_j_* in a sample, respectively, and R_ij_ is the absolute rank difference between the two lncRNAs within a sample. The mean[Rij(cancer)] represents the mean value of the absolute rank differences of the reversal lncRNA *L_i_* and *L_j_* in CRC tissues. Meanwhile, mean[Rij(non-cancer)] represents the mean value of absolute rank differences of the reversal lncRNA pair *L_i_* and *L_j_* in non-cancer tissues. Obviously, the higher the reversal degree for an lncRNA pair, the larger the reversal degree of the gene pair will be calculated between CRC and non-CRC samples.

All reversal lncRNA pairs between the CRC and non-CRC samples were identified in the training data, and *m* was denoted as the total number of the reversal lncRNA pairs. Then, the reversal lncRNA pairs were ranked in a descending order based on the reversal degree of each lncRNA pair. A forward selection procedure was further used to search optimal subsets of the reversal lncRNA pairs as a diagnosis biomarker. Namely, the top *k* (*k*=1…*m*) reversal lncRNA pairs were selected as a biomarker and the half-voting rule was used for classification. A sample would be classified to be a cancer sample if at least half of the biomarker lncRNA pairs showed the same REOs with cancer; otherwise, it would be classified to be a non-cancer sample. When the geometric mean of sensitivity and specificity in the training data reaches the highest, the value of *k* is selected. The most reversed *k* lncRNA pairs were selected as a candidate early diagnostic biomarker of CRC. Sensitivity and specificity were calculated as follows:


$$\text{Sensitivity}=\frac{TP}{\text{TP}+\text{FN}}$$

$$\text{Specificity}=\frac{\text{TN}}{\text{TN}+\text{FP}}$$

Here, cancer samples were defined as positive samples, and non-cancer samples were defined as negative samples. The TP, TN, FP, and FN represented the number of true-positive, true-negative, false-positive, and false-negative samples, respectively. Specially, to correctly classify CRC-adjacent normal tissues that are not accurately sampled as CRC, the CRC samples used to identify signatures include cancer samples and cancer-adjacent samples.

### RNA extraction and quantitative real-time polymerase chain reaction

The 75 samples from 35 CRC patients were retrieved from the Department of Pathology, Fudan University Shanghai Cancer Center (Shanghai, China). Clinical information and pathologic features were obtained from the medical record and pathology report. Total RNA was isolated from all fresh- frozen CRC tissues using a TRIzol reagent (Invitrogen, Carlsbad, CA, USA). Complementary DNA (cDNA) was synthesized with the Reverse Transcriptase Kit (Takara, # RR036A). Then, qPCR was performed using TB Green according to a standard protocol (Takara, #RR420A). Actin served as internal control. The primer pairs of lncRNAs used for qPCR are listed in **Supplementary Table S1**.

### Functional analysis of the diagnostic long non-coding RNA biomarkers

To further investigate the functions of the biomarker lncRNAs in CRC, the coexpressed mRNAs and the competing endogenous mRNAs were used to perform pathway enrichment analysis. On one hand, the top 500 mRNAs coexpressed with nine lncRNA biomarkers in the TCGA CRC dataset were taken as a candidate gene set. On the other hand, an lncRNA–mRNA network was constructed based on a competing endogenous RNA (ceRNA) hypothesis (19). Firstly, miRNA–lncRNA interactions were downloaded from the StarBase database (20). miRNA–mRNA interactions were obtained from miRBase (21). LncRNA–miRNA–mRNA relations documented in LncATCdb were also extracted (22). If an lncRNA and mRNA were regulated by an miRNA, then the lncRNA was connected in the lncRNA–mRNA network. By doing this, an lncRNA–mRNA network was constructed with 196,442 interactions between 14,039 mRNAs and 2,945 lncRNAs. Then, the interacted mRNAs with the biomarker lncRNAs were extracted from the constructed network as competing endogenous RNAs. Finally, the coexpressed mRNAs and the competing endogenous mRNAs were used to perform pathway enrichment analysis using R package clusterProfiler (23).

### Immune infiltration cell analysis

In order to investigate the association between the immune infiltration and the expression of the diagnostic lncRNA biomarkers, we calculated the immune cell infiltration among the cohort of TCGA CRC patients by CIBERSORT calculation (24). The Spearman correlation analysis was performed between the expression values of each biomarker lncRNA and the infiltration fractions of the immune cells, and the correlation coefficient R-values and significant P-values were calculated.

### Driver gene mutations and the diagnostic biomarker long non-coding RNAs

To explore the driver gene mutations related to the expression of biomarker lncRNAs in CRC, we analyzed the top five recurrent mutated driver genes recorded in COSMIC (*APC*, *KRAS*, *TP53*, *BRAF*, and *PIK3CA*) (25). Then, the expression differences of the lncRNAs between the mutant group and the wild-type group of each driver gene were analyzed by the Wilcoxon signed rank test using the TCGA CRC dataset.

## Results

### Identification of the diagnostic long non-coding RNA pair biomarker of colorectal cancer

**Figure 1:**
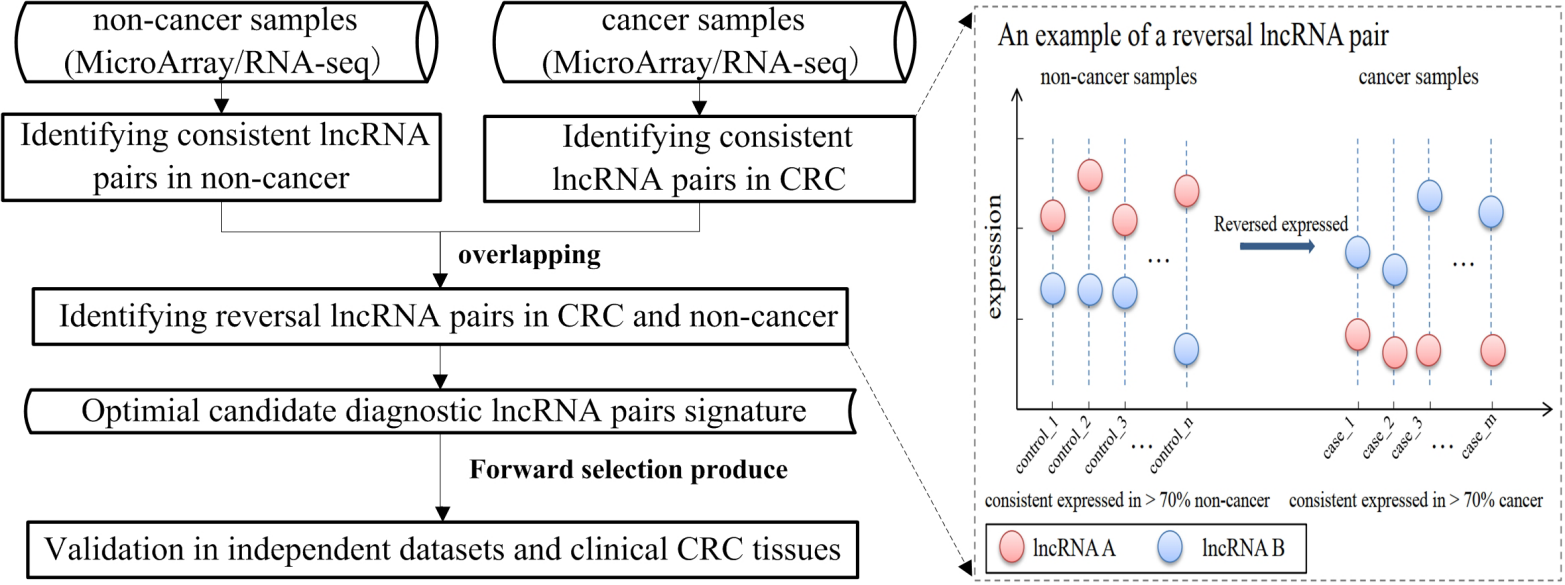
The workflow of identifying candidate diagnostic biomarker of colorectal cancer (CRC).

**Figure 2:**
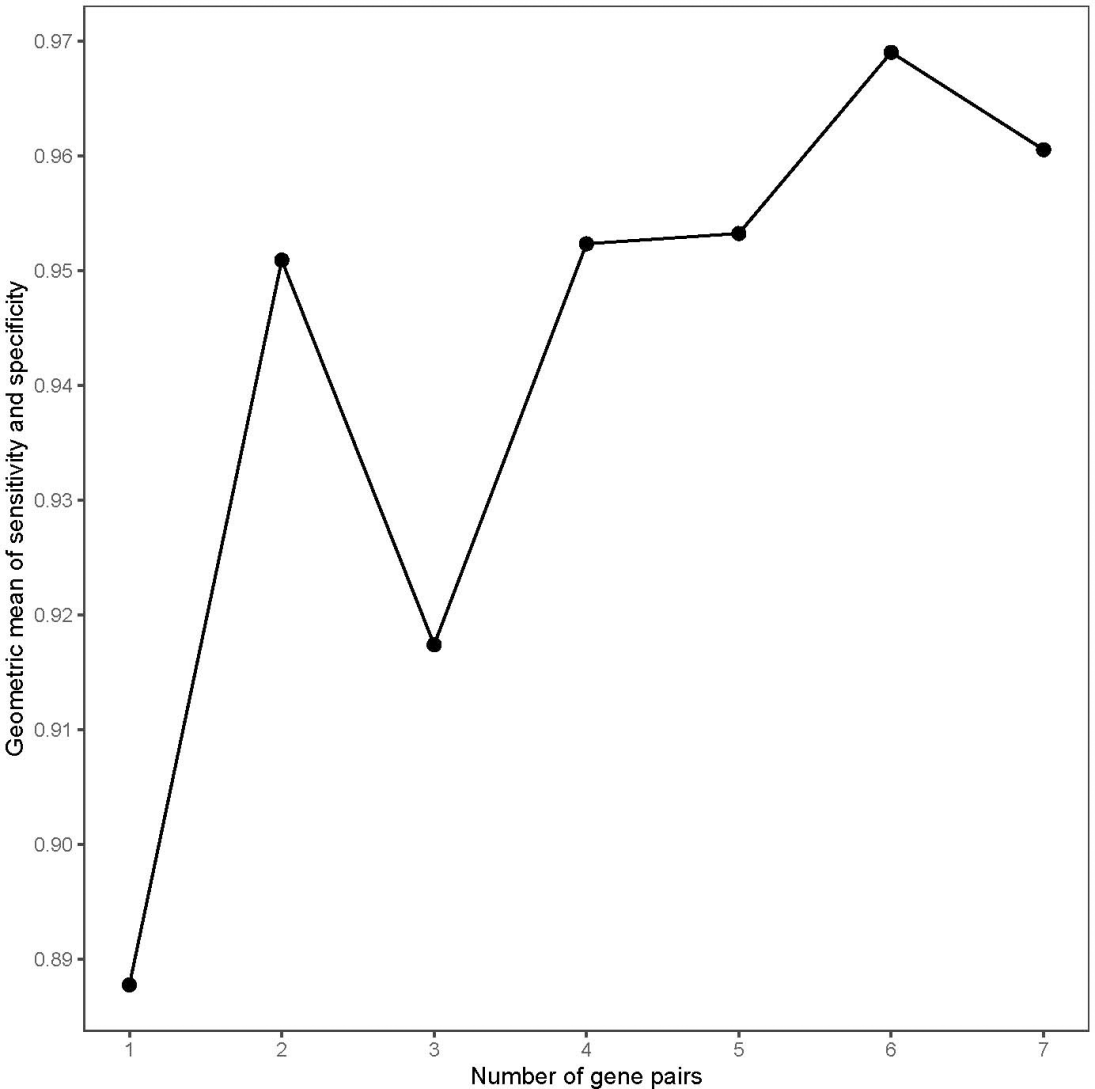
The geometric mean of the sensitivity and specificity of the top long non-coding RNA (lncRNA) pairs in the training data.

The workflow of this work is shown in **Figure 1**. Firstly, the cross-platform-consistent lncRNA pairs in non-cancer samples were detected. For the microarray platform, 160,732 consistent lncRNA pairs were identified in 32 samples of the GSE8671. For the RNA-seq platform, due to the imbalanced number of non-cancer samples between GSE76987 (20 samples) and GTEX (155 samples), we first identified consistent lncRNA pairs in each of the two datasets, and the 120,536 overlapping consistent lncRNA pairs were defined as consistent lncRNA pairs of the RNA-seq platform. Then, a total of 76,239 consistent lncRNA pairs overlapped in both microarray and RNA-seq platforms were defined as the cross-platform-consistent lncRNA pairs of non-cancer samples. Next, the cross-platform-consistent lncRNA pairs in cancer samples were detected. For the microarray platform, 16,884 consistent lncRNA pairs were identified in 40 samples of the GSE117606. For the RNA-seq platform, due to the similar sample size of different datasets, we pooled cancer samples into one dataset, and named it as RNA-seq_CRC (60 samples). Cancer-adjacent samples were also pooled into one dataset, and it was named as RNA-seq_Adjacent (40 samples). We identified 158,242 and 162,678 consistent lncRNA pairs for RNA-seq_CRC and RNA-seq_Adjacent, respectively, and 131,699 overlapping consistent lncRNA pairs were obtained for the RNA-seq platform. Overall, a total of 86,424 cross-platform-consistent lncRNA pairs of cancer samples were obtained for both microarray and RNA-seq platforms. Finally, seven cross-platform reversal lncRNA pairs were detected between CRC and non-cancer tissues. Then, according to the forward selection procedure and the half-voting rule described in the Materials and methods section, six lncRNA pairs were defined as the diagnostic biomarker in CRC. Further analysis showed that the relative expression levels of six lncRNA pairs of non-cancer tissues were significantly reversed in CRC tissues (Fisher’s exact test, P<2.2e-16, **Table S2**). With this biomarker, the geometric mean of the sensitivity and specificity could achieve 0.97, in which the sensitivity was 0.95 and the specificity was 0.98 (**Figure 2**). The six candidate lncRNA pairs are listed in **Table 2**. If at least three of the six lncRNA pairs showed that the expression level of gene A was less than gene B (**Table 2**), the tested sample was predicted as cancer. Otherwise, it was predicted as non-cancer.

**Table 2:** Six candidate diagnostic long non-coding RNA (lncRNA) pairs for colorectal cancer (CRC). In each lncRNA pair, gene B always has a higher expression level than gene A in CRC tissue samples compared with non-CRC tissue samples. If at least three of the six lncRNA pairs showed that the expression level of gene A is less than gene B, the tested sample was predicted as cancer.

lncRNA Pair	Gene A Symbol (Ensembl ID)	Gene B Symbol (Ensembl ID)
1	*LOC100130691 (ENSG00000213963)*	*LOC441204 (ENSG00000214870)*
2	*ZNF232-AS1(ENSG00000234327)*	*TOB1-AS1 (ENSG00000229980)*
3	*ZNF232-AS1(ENSG00000234327)*	*COPB2-DT (ENSG00000248932)*
4	*ZNF232-AS1(ENSG00000234327)*	*LINC01547 (ENSG00000183250)*
5	*ZNF232-AS1(ENSG00000234327)*	*ZNF22-AS1 (ENSG00000165511)*
6	*POLR2J4 (ENSG00000214783)*	*ZNF503-AS2 (ENSG00000237149)*

### Validation of the diagnostic long non-coding RNA pair biomarker in independent dataset

For a total of 950 CRC samples and 247 non-cancer samples, both of the sensitivity and specificity could achieve approximately 90% (**Table 3**). For CRC samples, 94% (89/95) and 89% (520/585) samples were correctly classified as cancer in the microarray platform and RNA-seq platform, respectively. For cancer-adjacent tissue samples, 97% (163/168) and 78% (80/102) of cancer-adjacent tissue samples were classified as cancer in the microarray platform and RNA-seq platform, respectively, indicating that the marker was also effective for most of tissue sampling in inaccurate locations. For adenoma samples, 84% (27/32) and 67% (42/62) of adenoma samples were classified as non-cancer in the microarray platform and RNA-seq platform. This might be because adenoma is a precancerous lesion of CRC, and the samples diagnosed as cancer already have the molecular characteristics of cancer. To further examine the specificity of six lncRNA pairs, another three datasets including inflammatory bowel disease (IBD) and normal tissues from patients with IBD were tested (**Table 4**). For IBD tissues, 97% (120/124) were correctly classified as non-cancer. For normal tissues from IBD patients, 93% (14/15) were correctly classified as non-cancer. The total specificity could achieve 96%. The results showed that the lncRNA pair marker had high specificity. To further examine the performance of the six lncRNA pairs in different CRC stages, 458 samples contain staging information were obtained from the TCGA dataset. The results showed that 87% of 78 patients with stage I, 82% of 183 patients with stage II, 93% of 132 patients with stage III, and 89% of 65 patients with stage IV were correctly identified as CRC (**Table 5**). To explore whether the six lncRNA pairs are stable in different microsatellite status, 110 CRC samples with MSI information from TCGA were evaluated. Approximately 95% of 63 patients with an MSS (microsatellite-stable) status and 82% of 47 patients with MSI-H (microsatellite instability–high) were correctly identified as CRC (**Table 5**). Tumors in the right-sided and left-sided colon exhibit different molecular characteristics and histology (26). Thus, the performance of the six lncRNA pairs in different sites were also tested. The results showed that the accuracy of left sided and right sided were 91% (161/177) and 85% (168/198), respectively.

**Table 3:** The performance of the lncRNA pair biomarkers in the validation dataset. .

Platform	Dataset	Normal	Adenoma	Cancer	Cancer-adjacent	Specificity	Sensitivity
**RNA-seq**	GSE76987	–	62(0.67)	4(1)	–	0.67	1
	GSE83687	–	–	–	61(0.77)	–	0.77
	GTEX	153(1)	–	–	–	1	–
	GSE107422	–	–	110(0.97)	–	–	0.97
	TCGA	–	–	471(0.87)	41(0.80)	1	–
**RNA-seq Total**		153(1)	62(0.67)	585(0.89)	102(0.78)	0.91	0.87
**Microarray**	GSE8671	–	32(0.84)	–	–	–	0.84
	GSE117606	–	–	34(1)	134(1)	–	1
	GSE35144	–	–	27(0.92)	–	–	–
	GSE22598	–	–	17(0.88)	17(0.88)	–	–
	GSE32323	–	–	17(0.88)	17(0.88)	–	–
**Microarray Total**		–	32(0.84)	95(0.94)	168(0.97)	0.84	0.96
**Total**		153(1)	94(0.72)	680(0.90)	270(0.90)	0.89	0.9

**Table 4:** The performance of the lncRNA pair biomarkers in independent inflammatory bowel disease and normal validation datasets. Accuracy is marked in parentheses.

Dataset	Platform	Normal	IBD	Specificity
GSE47908	MicroArray GPL570	15(0.93)	39(0.95)	0.94
GSE16879	MicroArray GPL570	–	61(0.97)	0.97
GSE14580	MicroArray GPL570	–	24(1)	1
Total		15(0.93)	124(0.97)	0.96

**Table 5:** Performance of the lncRNA pair biomarkers in different stages, the microsatellite instability status, and the site of CRC in the TCGA dataset.

	Status	Number of Samples	Accuracy
Stage	I	78	0.87
	II	183	0.82
	III	132	0.93
	IV	65	0.89
MSI Status	MSI	47	0.82
	MSS	63	0.95
Primary site	Left sided	177	0.913
	Right sided	198	0.853

### Validation of the diagnostic long non-coding pair biomarker using RT-qPCR (Quantitative real-time PCR)

To further validate the diagnostic lncRNA pair biomarker, A total of 75 samples form 35 CRC patients were enrolled to perform RT-qPCR, of which, cancer tissues and matched cancer-adjacent tissues were obtained from 30 patients, while 5 patients were took only cancer tissues. A total of 35 stage I–IV CRC patients were enrolled; the average age was 71.8 (range 35–80). A total of 20 patients were men, and 15 patients were women. The histology of all tumor specimens was moderately differentiated adenocarcinoma. All patients have undergone radical surgery for CRC (**Table 6**). In each lncRNA, two holes were established, and the mean value of two replicates was used. The detailed expression values are shown in **Table S3**. Based on the half-voting rule, one sample with at least three gene pairs showing the same relative expression level pattern with cancer is predicted as cancer. As a result, the accuracy of all 75 patients could achieve 93.3%. Through six lncRNA pair biomarkers, 32 of 35 (85.7%) CRC tissues and 28 of 30 (87.5%) adjacent tissues were correctly predicted as cancer by the six lncRNA pair biomarkers (**Table S5**). Specially, even in early stage (I and II), the accuracy could also achieve 90.9% (30/33). These results showed that the diagnostic lncRNA pair biomarker was highly sensitive in colorectal tissues.

**Table 6:** The characteristics of the CRC tissue samples.

SampleID	Gender	Age	TumorSize(cm)	TNM	Stage	TissueType
1	Male	60	5.5*4.5*1.8	T2N0M0	IIA	Tumor and Tumor-adjacent
2	Female	59	5*4.5*3	T4AN0M0	IIB	Tumor and Tumor-adjacent
3	Male	60	4*7*1.2	T3N0M0	IIA	Tumor and Tumor-adjacent
4	Male	54	7.5*4.5*1	T4AN0M0	IIB	Tumor and Tumor-adjacent
5	Female	44	3*2.5*1.7	T4N0M0	IIB	Tumor and Tumor-adjacent
6	Male	63	2.5*2*0.3	T2N0M0	IIA	Tumor and Tumor-adjacent
7	Female	56	3*2*0.8	T3N0M0	IIA	Tumor and Tumor-adjacent
8	Male	63	5.5*9.5*2	T3N0M0	IIA	Tumor and Tumor-adjacent
9	Male	54	1.8*2*0.8	T3N0M0	IIA	Tumor and Tumor-adjacent
10	Male	67	2.5*2*0.8	T3N0MX	IIA	Tumor and Tumor-adjacent
11	Male	64	3.5*3*0.8	T2N0MX	IIA	Tumor and Tumor-adjacent
12	Female	35	4*3.8*0.7	T3N0MX	IIA	Tumor and Tumor-adjacent
13	Male	55	3*2.5*1.8	T3AN0M0	IIA	Tumor and Tumor-adjacent
14	Female	51	4*3*2.5	T3N0M0	IIA	Tumor and Tumor-adjacent
15	Male	57	3.8*3.5*2	T3N0M0	IIA	Tumor and Tumor-adjacent
16	Male	60	4*3.8*1	T3N0MX	IIA	Tumor and Tumor-adjacent
17	Female	78	6.5*6*4	T3N0M0	IIA	Tumor and Tumor-adjacent
18	Female	48	3*3*1	T3N0M0	IIA	Tumor and Tumor-adjacent
19	Female	62	3.7*4*0.8	T3N0M0	IIA	Tumor and Tumor-adjacent
20	Female	51	8*8*2.5	T3N0M0	IIA	Tumor and Tumor-adjacent
21	Male	81	5.5*4*1	T3N0M0	IIA	Tumor and Tumor-adjacent
22	Female	57	3.5*3*0.5	T3N0MX	IIA	Tumor and Tumor-adjacent
23	Female	55	1.5*1.8*0.8	T3N0M0	IIA	Tumor and Tumor-adjacent
24	Female	69	3.5*3.5*2	T2N0M0	IIA	Tumor and Tumor-adjacent
25	Male	57	3.6*4.5*0.5	T3N0M0	IIA	Tumor and Tumor-adjacent
26	Female	67	2.5*2*1	T4N0M0	IIB	Tumor and Tumor-adjacent
27	Male	77	3.5*3*0.8	T3N0M0	IIA	Tumor and Tumor-adjacent
28	Male	75	5*4.5*2	T2N0MX	IIA	Tumor and Tumor-adjacent
29	Female	72	4*4*1.5	T3N0MX	IIA	Tumor and Tumor-adjacent
30	Male	75	2.5*1.5*1	T3N0M0	IIA	Tumor and Tumor-adjacent
31	Male	62	3.5*2.8*1	T4N0M0	II	Tumor
32	Male	69	7*6	T4NxM1	IV	Tumor
33	Male	73	4*3*1	T4N2aM0	IIIB	Tumor
34	Male	80	5*4*2	T2N0M0	I	Tumor
35	Female	75	2.5*2*1.5	T4N0M0	IIB	Tumor

### Exploring the functions of the biomarker long non-coding RNAs in colorectal cancer

**Figure 3:**
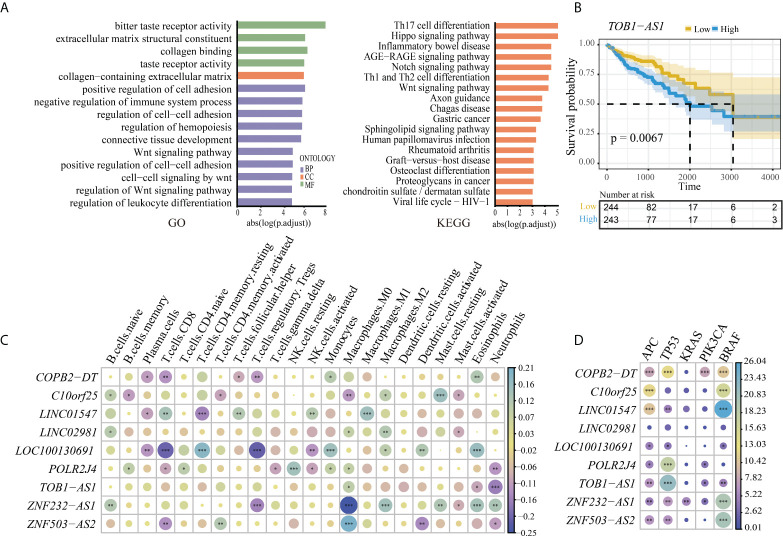
**(A)** The pathway enrichment analysis of the lncRNA biomarkers. Left is the result of Gene Ontology (GO); right is the result of KEGG. **(B)** High expression of lncRNA *TOB1-AS1* was associated with a worse prognosis outcome in CRC. **(C)** Association between the immune cell infiltration and the lncRNA biomarkers. *, **, and *** represent the P-values of 0.05, 0.01, and 0.001, respectively. The size of the circle represents the correlation coefficient. **(D)** The correlation of expression of lncRNA markers and driver genes in CRC. *, **, and *** represent the P-values of 0.05, 0.01, and 0.001, respectively. The size of the circle represents the size of the absolute value of log10 (P-value).

To further explore the functions of the six lncRNA pairs, 3,618 mRNAs obtained from coexpression or endogenous competition with the lncRNAs (**Table S4**) were used to perform pathway enrichment analysis. The enrichment analysis revealed that the lncRNAs were involved in highly associated cancer pathways such as cell–cell adhesion and Wnt signaling pathways and immune-associated pathways (**Figure 3A**). These findings convinced that our biomarker might play significant roles in the progression of cancer. The Kaplan–Meier survival analysis and log-rank test were also performed to test whether the lncRNAs were associated with the prognosis of CRC in the TCGA dataset. However, there was no significance between the expressions of these lncRNAs and the prognosis of CRC except *TOB1−AS1* (p = 0.0067, **Figure 3B**). To investigate the value of these lncRNA biomarkers in the tumor microenvironment, we analyzed 22 tumor immune cells using CIBERSORT and the output was visualized with a heatmap plot (**Figure 3C**). With a cutoff p-value less than 0.01, some lncRNAs tend to be associated with few types of immune cells. For example, *TOB1-AS1* was significantly negatively correlated with neutrophils. *LINC02981* was positively correlated with M2 macrophage. *POLR2J4* expression was significantly positively correlated with resting NK cells and negatively correlated with neutrophils. While some lncRNAs tend to be associated with multiple type of immune cells. *ZNF232-AS1* expression was positively correlated with M2 macrophage, eosinophils, neutrophils, resting mast cells, and naive B cells and negatively correlated with M0 macrophage and Treg. *LOC100130691* expression was positively correlated with resting memory CD4 T cells, monocytes, eosinophils, and activated dendritic cells and negatively correlated with T follicular helper cells, CD8 T cells, plasma cells, and activated NK cells. These results implied that these biomarker lncRNAs might be involved in the regulation of immune cell functions in CRC. The driver gene mutations related to the biomarker lncRNAs were also identified. *ZNF232-AS1* was significantly differentially expressed between the mutant group and the wild-type group for all five driver genes (**Figure 3D**). *TOB1-AS1* and *COPB2-DT* were significantly differentially expressed between the mutant group and the wild-type group for four of the five driver genes (*PIK3CA*, *APC*, *BRAF*, *TP53*). *ZNF503-AS2*, *POLR2J4*, *LOC100130691*, and *LINC01547* were significantly differentially expressed between the mutant group and the wild-type group for three of the five driver genes. *C10orf25* were significantly differentially expressed between the mutant group and the wild-type group of *APC* and *BRAF* genes. There was no significant difference for *LINC02981* expression for all driver genes.

## Discussion

In this study, we identified a robust biomarker of six lncRNA pairs for the correct diagnosis of CRC, which can discriminate CRC and CRC adjacent-normal tissues from non-cancer tissues. Regardless of platforms, the biomarker could achieve a sensitivity and specificity of 90%. Especially for the CRC samples and cancer-adjacent tissues, the accuracy could achieve ~90%. For adenoma samples, the accuracy could achieve 73%, which may be because adenoma is a precancerous lesion of CRC, and the samples diagnosed as cancer already have the molecular characteristics of cancer. Furthermore, the lncRNA pair biomarker showed excellent performance in all clinical stages; even in the early stage, the accuracy could achieve 87% and 82% in stage I and II. Meanwhile, the biomarker was also robust to the MSI status and primary tumor sites. These results further indicated that our biomarker is robust against clinicopathological variation.

In clinical practice, colonoscopy is the gold standard method and the most common used for early CRC screening. However, the miss rate of colonoscopy might be up to 8%. Most of these missed lesions were flat, non-polypoid growth-type cases (27). For such cases, the sampling location is often missed or inaccurate. For patients with clinical symptoms, multipoint random sampling can be used as a complement diagnostic method. In addition, indeterminate diagnosis is often met in pathology diagnosis in biopsy samples and highly relied on the experience of the pathologist. Misplaced epithelium in adenomas can occasionally be difficult to distinguish from invasive adenocarcinoma (7). In large sigmoid colonic adenomas, it is difficult to distinguish between the benign misplacement of the epithelium into the submucosa and invasive malignancy. This distinction requires a careful morphological evaluation of key differential features to determine the need for further endoscopic or surgical intervention, but there is little selective application of auxiliary immunohistochemistry (28). Sometimes, patients with IBD may lead to dysplastic changes, which also poses a challenge to the diagnosis of CRC (6). The six lncRNA pairs identified in our study provide a sensitive and robust measure for assisting the diagnosis of CRC. Moreover, the six lncRNA pairs (nine lncRNAs) based on the relative expression levels are robust to batch effects and could be applied to samples measured by either microarray, high-throughput RNA-seq or RT-qPCR. When the expression values of six lncRNA pairs were obtained, we could easily diagnose this sample by our marker. If at least three of the six lncRNA pairs showed that the expression level of gene A was less than that of gene B (**Table 2**), the tested sample was predicted as cancer. Otherwise, it was predicted as non-cancer.

The biomarker identified in this study consists of six lncRNA pairs from a set of nine lncRNAs, including *LOC100130691*, *LOC441204*, *ZNF232-AS1*, *TOB1-AS1*, *COPB2-DT*, *LINC01547*, *ZNF22-AS1*, *POLR2J4*, and *ZNF503-AS2*. Some of these lncRNAs may have played important roles in the initiation and progression of CRC as well as other tumors. It was reported that *POLR2J4* expression was significantly lower in the CRC samples compared with the normal samples, and it might play a significant role in the tumorigenesis of CRC. As the circularized product of *POLR2J4*, the knockdown of *circ_0079993* could significantly inhibit the proliferation of CRC cells *in vitro* (29). Furthermore, *LOC441204* activated by the β-Catenin/p21/*CDK4* pathway could promote the growth of tumor cells (30). Moreover, the overexpression of *TOB1-AS1* significantly inhibits cell proliferation, cell cycle progression, invasion, and induced apoptosis, while the knockdown of *TOB1-AS1* exhibits the opposite effect in both non-small cell lung cancer (31) and cervical cancer (32). In our study, *TOB1−AS1* was also found to be associated with the prognosis of CRC in the TCGA dataset. Le et al. found that the high expression of *LINC01547* is significantly related to worse overall survival in ovarian cancer patients (33). In addition, downregulated *LOC100130691* is associated with worse relapse-free survival and overall survival in patients with basal breast cancer (34). *ZNF503-AS2* was associated with a high risk of death in pediatric rhabdoid tumor of the kidney (35). The enrichment analysis revealed that the lncRNAs were involved in highly associated cancer pathways such as cell–cell adhesion and Wnt signaling pathway. Immune analysis showed that these marker lncRNAs were associated with multiple immune cells, implying that they might be involved in the regulation of immune cell functions in CRC. Most of the lncRNA biomarkers were also differentially expressed in the driver genes of CRC. These results implied that the biomarker lncRNAs were highly involved in CRC.

There are also some limitations in this study. Specificity and larger sample size need to be studied in the future. In addition, although the six lncRNA pairs performed excellently in normal and IBD tissues in the public validation set, PCR was not performed because the real normal samples (normal colorectal tissues from healthy people) were difficult to obtain.

In summary, we identified and validated six highly sensitive lncRNA pairs including nine lncRNAs as candidate biomarkers for aiding diagnosis of CRC.

## Data availability statement

The original contributions presented in the study are included in the article/**Supplementary Material**. Further inquiries can be directed to the corresponding authors.

## Ethics statement

This study was reviewed and approved by Shanghai Cancer Center Institutional Review Board, Shanghai Fudan University Cancer Center. The patients/participants provided their written informed consent to participate in this study.

## Author contributions

QY, HY and XZ conceived the idea and conceptualized the study. OW, QY and HY conducted the bioinformatics analysis and interpreted results. DS and YL conduct the RT-qPCR experiment. OW and YL collected and pre-processed data. QY wrote the paper and supervised the whole study process. QY, HY and DS revised the manuscript. All authors contributed to the article and approved the submitted version.

## Funding

This study was supported by Shanghai Sailing Program of Shanghai Science and Technology Commission (19YF1408500), Innovation Group Project of Shanghai Municipal Health Commission (2019CXJQ03), Shanghai Science and Technology Development Fund (19MC1911000), Shanghai Municipal Key Clinical Specialty (shslczdzk01301), and Innovation Program of Shanghai Science and technology committee (20Z11900300).

## Conflict of interest

The authors declare that the research was conducted in the absence of any commercial or financial relationships that could be construed as a potential conflict of interest.

## Publisher’s note

All claims expressed in this article are solely those of the authors and do not necessarily represent those of their affiliated organizations, or those of the publisher, the editors and the reviewers. Any product that may be evaluated in this article, or claim that may be made by its manufacturer, is not guaranteed or endorsed by the publisher.
