# Supplementary material for: lncRNA pair as candidate diagnostic signature for colorectal cancer based on the within-sample relative expression levels
Source: Front Oncol. 2022 Aug 17;12:912882. doi: 10.3389/fonc.2022.912882 (PMC9428707; doi:10.3389/fonc.2022.912882)
Supplement: Supplementary file 2 [file Table_2.docx]

**Table S2**. Comparisons of the relative expression between cancer and non-cancer samples.

| lncRNA Pair | Gene A Symbol | Gene B Symbol | Normal（A>B）ratio | Cancer（A>B）ratio | Pvalues |
| --- | --- | --- | --- | --- | --- |
| 1 | LOC100130691 | LOC441204 | 80.19% | 18.57% | 2.2e-16 |
| 2 | ZNF232-AS1 | TOB1-AS1 | 79.71% | 13.57% | 2.2e-16 |
| 3 | ZNF232-AS1 | COPB2-DT | 93.24% | 5.71% | 2.2e-16 |
| 4 | ZNF232-AS1 | LINC01547 | 97.10% | 15.00% | 2.2e-16 |
| 5 | ZNF232-AS1 | ZNF22-AS1 | 96.62% | 16.43% | 2.2e-16 |
| 6 | POLR2J4 | ZNF503-AS2 | 93.72% | 17.14% | 2.2e-16 |
